# Gastroprotective Effects of Nettle‐ and Carob‐Enriched Snail Mucus in a Rat Model of Gastric Injury: Role of Antioxidant and Anti‐Inflammatory Mechanisms

**DOI:** 10.1002/fsn3.72198

**Published:** 2026-08-03

**Authors:** Khaoula Gharbi, Slimen Selmi, Soumaya Wahabi, Hanen Kahlaoui, Karima Tlili, Stefano D'allacqua, Hichem Sebai

**Affiliations:** ^1^ Laboratory of Functional Physiology and Valorization of Bio‐Resources, Higher Institute of Biotechnology of Beja University of Jendouba Beja Tunisia; ^2^ Anaphate Service Tunis Military Hospital Tunis Tunisia; ^3^ Department of Pharmaceutical and Pharmacological Sciences University of Padova Padova Italy

**Keywords:** antioxidant activity, *Ceratonia siliqua*, gastric ulcer, gastroprotection, *Helix aspersa*, snail mucus, *Urtica dioica*

## Abstract

Dietary supplementation of *Helix aspersa Müller* with nettle ( *Urtica dioica* L.) or carob ( *Ceratonia siliqua* L.) has been hypothesized to enhance the biochemical composition and gastroprotective properties of the secreted mucus through enrichment of its polyphenolic, protein, and glycoprotein content. This study presents a comprehensive experimental evaluation of diet‐enriched snail mucus preparations across three supplementation levels (10%, 20%, and 30%) in an ethanol‐induced rat gastric ulcer model, encompassing phytochemical characterization, in vitro antioxidant assessment, and in vivo biochemical, systemic, and histopathological profiling. A standard snail mucus control group (SSSD) was included to isolate the specific contribution of dietary plant enrichment from the intrinsic properties of snail mucus itself. Analysis revealed that dietary supplementation significantly increased the yield of lyophilised snail mucus as well as its total phenolic, flavonoid, protein, and sugar contents in a dose‐dependent manner, with nettle exerting markedly superior effects than carob at all tested doses, attributable to its 33‐fold higher phenolic content and 15.5‐fold higher flavonoid content relative to carob extract. In vivo, pretreatment with enriched mucus preparations significantly attenuated ethanol‐induced gastric injury across all evaluated parameters. The 30% nettle‐enriched group (SSUD30%) demonstrated the strongest gastroprotective effect, normalizing gastric pH (3.75 ± 0.074), reducing gastric juice volume (1.59 ± 0.071 mL/100 g), restoring antioxidant enzyme activities (SOD, CAT, GPx) to near‐control values, reducing lipid peroxidation markers (MDA: 0.35 nmol/mg protein), and attenuating systemic inflammatory biomarkers including CRP, hepatic enzymes, and lipid profile alterations. Histopathological semi‐quantitative scoring confirmed a histological damage score reduction from 11 (ethanol group) to 2 in the SSUD30% group, corresponding to 81.82% protection, significantly exceeding that of famotidine (36.3%). These findings support a comprehensive gastroprotective model based on three complementary and interdependent mechanisms: reinforcement of the physical mucus barrier through increased viscosity and glycoprotein content, enhancement of endogenous antioxidant defenses through restoration of SOD, CAT, and GPx activities via the Nrf2/HO‐1 pathway, and attenuation of systemic inflammatory cascades through inhibition of NF‐κB‐mediated signaling by flavonoids and polyphenols incorporated into the enriched mucus. Overall, diet‐enriched snail mucus, particularly SSUD30%, should be conceptualized not merely as a topical mucoprotective agent, but as a multi‐target bioactive preparation whose efficacy depends on the complementary contribution of physical barrier reinforcement, antioxidant restoration, and anti‐inflammatory modulation. Future studies should prioritize identification of the specific bioactive compounds responsible for the observed effects, assessment of bioavailability under gastrointestinal conditions, and validation of therapeutic potential in human clinical settings.

## Graphical abstract

Dietary enrichment of *Helix aspersa* mucus with *Urtica dioica* or *Ceratonia siliqua* extracts increased its phenolic, flavonoid, protein, and glycoprotein content in a dose‐dependent manner, with nettle showing the strongest effect. In an ethanol‐induced rat model of gastric ulcer, the 30% nettle‐enriched mucus (SSUD30%) markedly reduced gastric lesions (81.82% protection vs. 36.3% for famotidine), normalized gastric pH, and restored antioxidant enzyme activities (SOD, CAT, and GPx) via the Nrf2/HO‐1 pathway while attenuating NF‐\u03baB‐driven inflammatory markers. These findings support a triple gastroprotective mechanism: reinforcement of the mucus barrier, restoration of antioxidant defenses, and attenuation of inflammatory signaling.

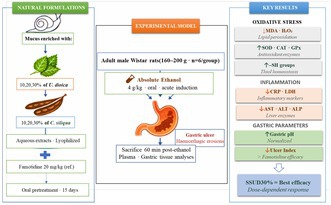

## 1 Introduction

Gastric ulcers represent a major gastrointestinal health burden, affecting an estimated 4 million individuals annually and frequently complicated by hemorrhage, perforation, or gastric outlet obstruction (Lanas and Chan 2017; Kavitt et al. 2019). Their etiology is multifactorial, reflecting a critical imbalance between mucosal‐damaging and cytoprotective factors. *Helicobacter pylori* infection remains the leading cause, implicated in 70%–80% of gastric ulcers and up to 90% of duodenal ulcers globally, inducing injury through chronic bacterial colonization and immune‐mediated mucosal inflammation (Hooi et al. 2017; Zamani et al. 2022). In contrast, non‐steroidal anti‐inflammatory drugs (NSAIDs) act through a distinct systemic mechanism: inhibition of cyclo‐oxygenase (COX) enzymes resulting in suppressed prostaglandin synthesis, impaired mucus and bicarbonate secretion, and reduced mucosal blood flow, accounting for 15%–25% of ulcer cases in developed countries (Lanas and Chan 2017; D'Arcy et al. 2014). Additional risk factors, including excessive alcohol consumption and poor dietary habits, further compromise mucosal integrity by amplifying oxidative stress (Gugliandolo et al. 2021).

At the molecular level, gastric injury is primarily driven by the overproduction of reactive oxygen species (ROS), which trigger lipid peroxidation, DNA damage, and epithelial cell death. Concomitantly, the transcription factor NF‐κB is activated and upregulates pro‐inflammatory mediators TNF‐α, IL‐6, IL‐1β, and COX‐2, perpetuating a damaging inflammatory loop (Tarnawski et al. 2013). In ethanol‐induced injury specifically, mucosal damage results from direct disruption of epithelial membranes, glutathione depletion, malondialdehyde (MDA) accumulation, and microvascular hemorrhage (Robert et al. 1979; Szabo et al. 1985). This model was selected for the present study given its well‐established reproducibility, rapid and quantifiable lesion induction, and accurate recapitulation of the oxidative‐inflammatory hallmarks of human gastric ulceration, advantages over chronic NSAID or *H. pylori* models in the context of preclinical gastroprotection research (Sairam et al. 2003).

Current pharmacological therapies proton pump inhibitors (PPIs), H2‐receptor antagonists, and antibiotic‐based *H. pylori* eradication regimens face growing limitations, including antibiotic resistance, high recurrence rates, and adverse effects associated with long‐term use such as gut dysbiosis and hypomagnesaemia (Sharma et al. 2021; Vakil 2022). These shortcomings have stimulated considerable interest in natural bioactive compounds as gastroprotective agents or therapeutic adjuvants (Gugliandolo et al. 2021).

Among emerging natural candidates, the mucus of the garden snail *Helix aspersa Müller* has attracted scientific attention due to its unique composition of allantoin, collagen, glycosaminoglycans, glycoproteins, and phenolic antioxidants (Brieva et al. 2008; Trapella et al. 2018). In vivo studies have confirmed its gastroprotective efficacy in ethanol‐induced ulcer models, demonstrating significant reduction in macroscopic lesion indices and attenuation of histological damage, with activity comparable to famotidine (Petrov et al. 2022). Its bioactive constituents also suppress NF‐κB‐mediated inflammatory pathways and promote mucosal regeneration through stimulation of fibroblast proliferation and epithelial turnover (Brieva et al. 2008). Key components, particularly allantoin and glycoproteins, retain sufficient biological activity under gastric acid conditions to exert cytoprotective effects at the mucosal level (Trapella et al. 2018). Regarding safety, *H. aspersa* mucus has a well‐established tolerability profile in dermatological applications, with rodent toxicity studies reporting no significant adverse effects at therapeutic doses; however, formal gastrointestinal safety data remain limited and warrant further investigation (Brieva et al. 2008; Petrov et al. 2022).

*Urtica dioica* L. (nettle) is rich in quercetin, kaempferol, and phenolic acids, which scavenge ROS, inhibit lipid peroxidation, and suppress NF‐κB/COX‐2/TNF‐α signaling (Khan et al. 2020). *Ceratonia siliqua* L. (carob) provides condensed tannins and gallic acid derivatives that inhibit gastric acid secretion and promote mucosal healing (Almeida et al. 2021). When incorporated into the snail diet, these plant‐derived polyphenols are metabolized and accumulated within the mucus, enriching its bioactive profile (Petrov et al. 2022). The combination is hypothesized to produce a synergistic rather than merely additive effect: the anti‐secretory and mucosal‐coating actions of carob tannins complement the antioxidant and anti‐inflammatory properties of nettle flavonoids, whereas the tissue‐regenerative components of snail mucus address a third mechanistic dimension not covered by either plant extract alone, forming a pharmacologically rational, multi‐target gastroprotective strategy.

To our knowledge, this is the first study to evaluate whether dietary biofortification of *H. aspersa Müller* with *U. dioica* L. or *C. siliqua* L. modulates the gastroprotective efficacy of the resulting enriched mucus in an ethanol‐induced rat ulcer model. Unlike studies evaluating snail mucus or plant extracts in isolation, the present work introduces a biogenic enrichment strategy to generate a compositionally enhanced secretion with augmented therapeutic potential. Specifically, this study aimed to: (1) characterize the phytochemical profile and antioxidant capacity of enriched mucus preparations at three supplementation levels (10%, 20%, 30%); (2) evaluate gastroprotective efficacy through ulcer index, gastric juice analysis, and biochemical profiling of oxidative stress and antioxidant enzyme markers in gastric tissue; (3) assess systemic safety through plasma biomarker analysis; and (4) confirm histopathological outcomes through semi‐quantitative mucosal scoring.

## 2 Materials and Methods

### 2.1 Chemicals

Reduced glutathione (GSH), ethylenediaminetetraacetic acid (EDTA), thiobarbituric acid (TBA), 2,6‐di‐tert‐butyl‐4‐hydroxytoluene (BHT), sodium carbonate (Na_2_CO_3_), trichloroacetic acid (TCA), hydrogen peroxide (H_2_O_2_), potassium dihydrogen phosphate (KH_2_PO_4_), bovine catalase, epinephrine, 2,4‐dinitrophenylhydrazine (DNPH), hydrochloric acid (HCl), sodium hydroxide (NaOH), sodium chloride (NaCl), Folin–Ciocalteu reagent, aluminium chloride (AlCl_3_), and 2,2‐diphenyl‐1‐picrylhydrazyl (DPPH) were obtained from Sigma‐Aldrich (St. Louis, MO, USA). All chemicals were of analytical grade unless otherwise stated.

### 2.2 Botanical Authentication, Preparation of Plant Extracts, and Snail Mucus

Plant materials were authenticated by Professor Chokri Hafsi (University of Jendouba, Tunisia), a qualified botanist. Aerial parts of *U. dioica* L. (voucher specimen No. SO.325) and pods of *C. siliqua* L. were collected from the Kef region (northwestern Tunisia). The botanical identity of both species was confirmed by the same expert. Voucher specimens were deposited in the Herbarium of the Higher Institute of Biotechnology of Béja, University of Jendouba, in accordance with previous work from our laboratory (Rtibi et al. 2015), and are available under the corresponding reference numbers.

Preparation of aqueous plant extracts: plant materials were dried in a forced‐air oven at 40°C for 7 days and finely ground using a laboratory blender. To obtain aqueous extracts, 10 g of each dried powder was suspended in 100 mL of distilled water and agitated on an orbital shaker at 150 rpm and 25°C for 24 h. The suspensions were then filtered through Whatman No. 1 filter paper, frozen at −20°C, and lyophilised using a freeze‐dryer (Christ Alpha 1–4, Germany). The extraction yield was calculated gravimetrically as the mass of lyophilised extract obtained relative to the initial dry plant material and expressed as percentage (% w/w). The lyophilised aqueous extracts of *U. dioica* (AEUD) and *C. siliqua* (AECS) were reconstituted in distilled water at appropriate concentrations for phytochemical analyses and in vivo administration.

Preparation of diet‐enriched snail mucus: A total of 160 *H. aspersa* snails, aged 3 months and weighing 16 ± 2 g, were acclimatized for 1 week under controlled conditions (humidity, 52%; daytime temperature, 16°C; nighttime temperature, 8°C; 12 h photoperiod) before diet assignment. Snails were randomly allocated into seven groups of 20 individuals each and maintained in polypropylene cages.

Experimental groups received diets supplemented with nettle or carob powder at three inclusion levels, together with basal concentrate and calcium carbonate, as follows: SSUD10% (60% concentrate, 10% nettle, 30% CaCO_3_), SSUD20% (50% concentrate, 20% nettle, 30% CaCO_3_), SSUD30% (40% concentrate, 30% nettle, 30% CaCO_3_), SSCS10% (60% concentrate, 10% carob, 30% CaCO_3_), SSCS20% (50% concentrate, 20% carob, 30% CaCO_3_), and SSCS30% (40% concentrate, 30% carob, 30% CaCO_3_).

An additional group received a standard diet consisting of 70% concentrate and 30% CaCO_3_ (SSSD group). A control group received the basal diet without supplementation.

After 8 weeks of dietary supplementation, mucus was collected by gentle mechanical stimulation of the foot. The collected mucus was immediately frozen at −80°C and lyophilised. The extraction yield of snail mucus was calculated as the ratio of the lyophilised mucus weight to the initial fresh mucus weight collected from each group and expressed as percentage (% w/w). The lyophilised mucus yield from each dietary group was recorded, and the powder was reconstituted in distilled water at 25 mg/mL for in vivo administration.

### 2.3 Animals, Ethical Approval, and Experimental Design

All animal procedures were approved by the Medical Ethical Committee for the Care and Use of Laboratory Animals of the Pasteur Institute of Tunis (Approval No. LNFP/Pro 152012). Adult male Wistar rats (160–200 g; 8 weeks old) were obtained from the Biotechnology Institute of Béja, Tunisia. Animals were housed in groups of six per polypropylene cage under controlled conditions (22°C ± 2°C, 50%–60% relative humidity, 12 h light–dark cycle) with free access to standard pellet diet and tap water during the 1‐week acclimatization period.

The oral dose of snail mucus (25 mg/kg body weight) was selected based on previously published gastroprotective studies using *H. aspersa* mucus in rodent models (Petrov et al. 2022) and on preliminary tolerance observations in our laboratory, in which no signs of acute toxicity were observed at this dose.

The ethanol‐induced gastric ulcer model was chosen because of its reproducibility, rapid lesion induction, and ability to reproduce key oxidative and inflammatory features of gastric mucosal injury within a short experimental timeframe. Compared with chronic NSAID‐ or *H. pylori* ‐associated models, the ethanol model allows acute and standardized mucosal injury, making it suitable for evaluating short‐term gastroprotective interventions (Robert et al. 1979; Szabo et al. 1985).

Animals were divided into 10 groups. Group 1 served as the normal control and received water only. Group 2 served as the ethanol control. Groups 3 received snail mucus at 25 mg/kg body weight from snails fed the standard diet (SSSD); Groups 4–6 received snail mucus at 25 mg/kg body weight from snails fed 10% nettle (SSUD10%), 20% nettle (SSUD20%), and 30% nettle (SSUD30%), respectively. Groups 7–9 received snail mucus at 25 mg/kg body weight from snails fed 10% carob (SSCS10%), 20% carob (SSCS20%), and 30% carob (SSCS30%), respectively. Group 10 received famotidine (20 mg/kg body weight, orally) for 15 days.

After 15 days of pretreatment, animals were fasted for 24 h with free access to water. Two hours after the final treatment, Groups 2–10 received absolute ethanol (4 g/kg body weight) by oral gavage to induce acute gastric ulceration. Group 1 received an equivalent volume of distilled water. Animals were sacrificed by cervical dislocation 60 min after ethanol administration. Blood was collected by cardiac puncture into heparinised tubes and centrifuged at 3000 × *g* for 15 min at 4°C. Plasma was separated and stored at −80°C until biochemical analysis. Gastric tissues were immediately excised for macroscopic and biochemical assessment.

### 2.4 Determination of Total Phenol Content

Total phenolic content (TPC) was determined using the Folin–Ciocalteu method as described by Singleton et al. (1999). Briefly, 500 μL of each sample was mixed with 10 mL of distilled water and 0.5 mL of Folin–Ciocalteu reagent (1:1, v/v). After 5 min at 25°C ± 1°C, 8 mL of 7.5% (w/v) Na_2_CO_3_ was added. The reaction mixture was incubated for 2 h in the dark, and absorbance was measured at 765 nm using a UV–Vis spectrophotometer. A calibration curve was prepared with gallic acid (0–100 mg/L; *R* ^2^ ≥ 0.998), and results were expressed as mg gallic acid equivalents per gram of dry matter (mg GAE/g DM). All measurements were performed in triplicate.

### 2.5 Determination of Total Flavonoid Content

Total flavonoid content (TFC) was measured using the aluminium chloride colorimetric assay adapted from Chang et al. (2002). One milliliter of each extract was mixed with 1 mL of 2% AlCl_3_ in methanol and incubated for 15 min at 25°C in the dark. Absorbance was read at 430 nm against a methanol blank. A calibration curve was prepared using quercetin (10–100 μg/mL; *R* ^2^ ≥ 0.998), and results were expressed as mg quercetin equivalents per gram of dry matter (mg QE/g DM). All measurements were performed in triplicate.

### 2.6 Determination of Total Sugars

Total sugars were quantified using a modified phenol‐sulfuric acid method (Li et al. 2021). Briefly, 1 mL of sample was mixed with 50 μL of 75% phenol, followed by rapid addition of 2.5 mL of concentrated sulfuric acid. The mixture was incubated for 30 min at room temperature in the dark, and absorbance was measured at 485 nm. Total sugar content was calculated from a glucose standard curve and expressed as mg glucose equivalents per gram of dry matter (mg GluE/g DM).

### 2.7 Protein Determination

Protein content was determined using the Coomassie Brilliant Blue G250 method (Kruger 1994). The assay is based on the binding of the dye to basic amino acid residues, resulting in a shift in absorbance from 465 to 595 nm. Absorbance was measured at 595 nm, and protein concentration was determined from a bovine serum albumin (BSA) calibration curve.

### 2.8 Evaluation of Antioxidant Activity by DPPH Radical Scavenging Assay

Free radical scavenging activity of the extracts and mucus preparations was assessed using the DPPH assay as described by Kumar et al. (2018). Serial dilutions of each sample were mixed with an equal volume of 0.1 mM DPPH solution in methanol. After 30 min of incubation in the dark at 25°C, absorbance was measured at 517 nm. Radical scavenging activity was calculated as % inhibition = [(*A*_control − *A*_sample)/*A*_control] × 100. The IC_50_ value was determined from the dose–response curve by nonlinear regression. Quercetin was used as the positive control. All measurements were performed in triplicate.

### 2.9 Macroscopic Evaluation of Gastric Mucosal Lesions

The stomach was removed and opened along the greater curvature. The tissue was gently rinsed with 0.9% saline to remove debris. Gastric mucosal lesions were visually inspected, and photographs of haemorrhagic erosions were taken for documentation. The ulcer index was calculated by summing the lengths of all gastric lesions. Lesion scoring was performed by two independent observers blinded to group allocation.

### 2.10 Measurement of Gastric Juice pH and Volume

After macroscopic examination, gastric contents were collected into pre‐weighed tubes and centrifuged at 2000 × *g* for 10 min to remove debris. The volume of clear gastric juice was measured and expressed as mL per 100 g body weight (Konturek and Konturek 2004). Gastric juice pH was measured immediately using a calibrated digital pH meter (accuracy ±0.01 pH units) at 25°C. Both parameters were assessed in duplicate for each animal.

### 2.11 Preparation of Gastric Mucosal Homogenates

Following macroscopic assessment, gastric mucosal tissue was carefully scraped from the underlying muscularis layer and homogenized (1:10, w/v) in ice‐cold phosphate buffer (KH_2_PO_4_/K_2_HPO_4_, 50 mM, pH 7.4) using a high‐speed homogenizer (10,000 rpm, 3 × 30 s on ice). Homogenates were centrifuged at 10,000 × *g* for 10 min at 4°C, and the supernatants were collected and stored at −80°C for subsequent biochemical analyses. Total protein concentration was determined using the Bradford assay (Bradford 1976) with BSA as the standard. All enzymatic and oxidative stress parameters were normalized to total protein content.

### 2.12 Evaluation of Plasma Biomarkers

Plasma biomarkers were measured to assess the systemic effects of ethanol toxicity, which extend beyond local gastric injury and may involve hepatocellular stress, pancreatic enzyme release, dyslipidaemia, and renal impairment in rodent models (Zhang et al. 2019; Lee et al. 2023). The following parameters were measured using an automated biochemical analyzer (SELECTRA PRO XL; ELI Tech Group Clinical System SAS, Tunisia) with validated commercial kits: hepatic markers (AST, ALT, and ALP), lipid profile (LDL‐C and HDL‐C), renal markers (urea and creatinine), pancreatic function (amylase), and markers of systemic inflammation and tissue damage (CRP and LDH). All assays were performed in duplicate, and results are expressed in SI units.

### 2.13 Lipid Peroxidation Measurement (MDA) in Gastric Mucosal Tissue

Lipid peroxidation in gastric mucosal homogenates was quantified by measuring malondialdehyde (MDA) using the thiobarbituric acid reactive substances assay, adapted from Ohkawa et al. (1979). Homogenate aliquots (200 μL) were treated with 1% BHT in 20% TCA to prevent ex vivo oxidation and centrifuged at 1000 × *g* for 5 min at 4°C. The supernatant was reacted with 0.5 N HCl and 120 mM thiobarbituric acid in Tris buffer (pH 7.4) at 80°C for 10 min to form the MDA‐TBA chromophore. Absorbance was measured at 532 nm, and MDA concentrations were calculated using a molar extinction coefficient of 1.56 × 10^5^ M^−1^ cm^−1^. Results are expressed as nmol MDA per mg protein.

### 2.14 Determination of Hydrogen Peroxide (H_2_O_2_ ) in Gastric Mucosal Tissue

Hydrogen peroxide levels in gastric mucosal homogenates were determined spectrophotometrically using a peroxidase‐catalyzed colorimetric assay based on the oxidation of p‐hydroxybenzoic acid and 4‐aminoantipyrine to form a pink quinoneimine chromophore (Zhang et al. 2019). Homogenates were incubated in reaction buffer (0.1 M phosphate buffer, pH 7.4) containing 0.5 mM p‐hydroxybenzoic acid, 0.3 mM 4‐aminoantipyrine, and 1 U/mL horseradish peroxidase at 37°C for 30 min. Absorbance was read at 505 nm against a standard curve of known H_2_O_2_ concentrations (0–100 μM; detection limit, 0.1 μM). Results are expressed as μmol H_2_O_2_ per mg protein.

### 2.15 Assays for Antioxidant Enzyme Activity

Superoxide dismutase (SOD) activity was assayed by the epinephrine autoxidation method (Sun and Zigman 1978), which measures inhibition of adrenochrome formation at 480 nm in sodium carbonate–bicarbonate buffer (62.5 mM, pH 10.2). One unit of SOD was defined as the amount causing 50% inhibition of epinephrine autoxidation. Catalase (CAT) activity was determined by monitoring H_2_O_2_ decomposition at 240 nm (*ε* = 43.6 M^−1^ cm^−1^) in 50 mM phosphate buffer (pH 7.0) containing 15 mM H_2_O_2_ (Aebi 1974), and expressed as μmol H_2_O_2_ decomposed per min per mg protein. Glutathione peroxidase (GPx) activity was measured using the coupled enzymatic assay of Flohé and Günzler (1984), monitoring NADPH oxidation at 340 nm (*ε* = 6.22 mM^−1^ cm^−1^); activity was expressed as nmol NADPH oxidized per min per mg protein. All assays were conducted in triplicate at 25°C using freshly prepared reagents and appropriate blanks.

### 2.16 Determination of Total Sulfhydryl Group Concentration in Gastric Mucosal Tissue

Total sulfhydryl (−SH) content in gastric mucosal homogenates was determined using Ellman's reagent (DTNB) according to Ellman (1959). Aliquots of 100 μL homogenate were mixed with 900 μL of 0.1 M Tris–HCl buffer (pH 8.2) containing 20 mM EDTA. Initial absorbance (*A*_1_) was recorded at 412 nm. The reaction was initiated by adding 100 μL of 10 mM DTNB in absolute methanol, followed by incubation for 15 min at 25°C in the dark. Final absorbance (*A*_2_) was measured at 412 nm, and sulfhydryl concentration was calculated as [−SH] = (*A*_2_ – *A*_1_ − *B*) × 1.57, where 1.57 mM^−1^ cm^−1^ is the molar extinction coefficient of the TNB^2−^ anion. Results are expressed as μmol −SH groups per mg protein.

### 2.17 Histopathological Analysis With Semi‐Quantitative Scoring

Gastric tissue samples were collected from the glandular region of the stomach immediately after sacrifice, rinsed in 0.9% NaCl, and fixed in 10% neutral‐buffered formalin for 24 h. Fixed tissues were dehydrated through a graded ethanol series, cleared in xylene, and embedded in paraffin. Serial sections (5 μm) were cut using a rotary microtome, deparaffinized, rehydrated, and stained with hematoxylin and eosin (HetE). Stained sections were examined under light microscopy (×100 and ×400) by two independent trained observers blinded to group allocation. A semi‐quantitative histological damage scoring system, adapted from Lacy and Ito (1982) and modified for gastric mucosal assessment, was applied on a scale of 0–3:

Normal mucosa: intact epithelium, no oedema, no inflammatory infiltration.

Mild damage: superficial epithelial erosion with minimal inflammatory cell infiltration.

Moderate damage: submucosal edema, focal hemorrhage, and moderate leukocyte infiltration.

Severe damage: extensive mucosal destruction, diffuse hemorrhage, and dense inflammatory infiltration.

The total histological damage score (HDS) for each animal was calculated as the sum of individual parameter scores (maximum score: 12).

### 2.18 Statistical Analysis

All results are presented as mean ± standard error of the mean (SEM). Group comparisons were performed using SPSS software version 20, employing one‐way analysis of variance (ANOVA) followed by Tukey's post hoc test. Statistical significance was set at *p* < 0.05.

## 3 Results

### 3.1 Extraction Yield of Plant Extracts and Snail Mucus

The extraction yield of the aqueous plant extracts and snail mucus is presented in Table 1. A clear difference was observed between the two plant species, with *U. dioica* extract (AEUD) showing a higher yield (12.18% ± 0.97% w/w) compared to *C. siliqua* extract (AECS) (9.47% ± 1.08% w/w), indicating a greater recovery of soluble dry matter under identical extraction conditions. Regarding snail mucus, the lyophilised mucus yield was significantly influenced by dietary supplementation. The lowest yield was recorded in the standard diet group (SSSD) (1.20% ± 0.07% w/w), whereas all supplemented groups exhibited increased yields in a dose‐dependent manner. The highest values were observed in the SSUD30% group (2.82% ± 0.06% w/w) and SSCS30% group (2.51% ± 0.01% w/w), with nettle supplementation producing a more pronounced effect than carob at equivalent doses. Overall, these findings suggest that dietary plant supplementation modulates the recovery of dry matter in snail mucus, reflecting both changes in secretion and/or compositional enrichment of the mucus matrix.

**TABLE 1:** Yield, total proteins, sugars, polyphenol and flavonoid content.

	Yield (% w/w)	Proteins (mg/g dry matter)	Sugars (mg/g dry matter)	Total phenolic content (mg GAE/g)	Total flavonoids content (mg QE/g)	DPPH IC_50_ (μg/mL)
AEUD	12.18 ± 0.97	23.66 ± 0.12	29.39 ± 0.061	218.5 ± 0.36	31.18 ± 1.22	24.62 ± 1.96
AECS	9.47 ± 1.08	8.31 ± 0.67	208.7 ± 0.092	6.52 ± 0.59	2.01 ± 0.47	323.16 ± 0.75
SSSD	1.2 ± 0.07	521 ± 1.57	2.62 ± 0.037^#^	0.33 ± 0.0047	0.52 ± 0.018	160.36 ± 0.32
SSUD10%	2.31 ± 0.01*^#^	605 ± 2.51*^#^	2.53 ± 0.058^#^	0.45 ± 0.061*^#^	0.71 ± 0.064*^#^	142.36 ± 0.26*^#^
SSUD20%	2.67 ± 0.009*^#^	615 ± 1.87*^#^	2.73 ± 0.87^#^	0.57 ± 0.012*^#^	0.88 ± 0.067*^#^	136.19 ± 0.088*^#^
SSUD30%	2.82 ± 0.06*^#^	644 ± 1.49*^#^	2.81 ± 0.035	0.64 ± 0.026*^#^	0.93 ± 0.082*^#^	131.81 ± 0.17*^#^
SSCS10%	1.9 ± 0.004*	597 ± 1.24*	4.42 ± 1.82*	0.35 ± 0.0074	0.54 ± 0.026	156.4 ± 0.13*
SSCS20%	2.32 ± 0.01*	580 ± 3.67*	4.86 ± 0.55*	0.36 ± 0.0091	0.57 ± 0.009	149.31 ± 0.21*
SSCS30%	2.51 ± 0.01*	568 ± 2.81*	5.12 ± 0.57*	0.41 ± 0.013*	0.63 ± 0.017*	141.55 ± 0.076*
Quercetin	/	/	/	/	/	58.36 ± 0.31

*Note:* As well as IC50 values from the DPPH free radical scavenging assay of the aqueous extract of nettle, carob, different types of snail mucus (fed with standard diet, 10%, 2% and 3% nettle and carob) and quercetin. The data are expressed as mean ± SEM (*n* = 6).

*p < 0.05 compared to SSSD and ^#^ *p* < 0.05 compared to SSCS.

### 3.2 Analysis of Phenolic Compounds in Extracts

The total phenolic content, summarized in Table 1, expressed as mg gallic acid equivalent per gram (mg GAE/g), demonstrates a marked difference between plant extracts, with nettle extract (AEUD) showing a 33‐fold higher content than carob extract (AECS) (218.5 ± 0.36 vs. 6.52 ± 0.59 mg GAE/g). This difference in plant composition is reflected in the phenolic composition of snail slime: the control group fed with a standard diet (SSSD) shows a content of 0.33 ± 0.005 mg GAE/g, whereas supplementation with nettle induces a significant and dose‐dependent increase in the phenolic content of the slime at all tested doses compared to the control (SSUD10% = 0.45 ± 0.061 mg GAE/g, SSUD20% = 0.57 ± 0.012 mg GAE/g, SSUD30% = 0.64 ± 0.026 mg GAE/g), with values also significantly higher than the corresponding groups supplemented with carob at equivalent doses. In contrast, carob supplements cause a more modest increase, with only the 30% dose reaching significance compared to the control (SSCS30% = 0.41 ± 0.013 mg GAE/g), whereas the 10% and 20% doses do not differ significantly from the control (SSCS10% = 0.35 ± 0.007 mg GAE/g, SSCS20% = 0.36 ± 0.009 mg GAE/g). The slime richest in phenolic compounds is that from snails supplemented with 30% nettle (SSUD30%), demonstrating that the phenolic composition of plant‐based diet directly influences the biochemical composition of slime secreted by *H. aspersa* , with a clearly superior effect of nettle compared to carob at all tested doses.

### 3.3 Analysis of Flavonoid Compounds in Extracts

The total flavonoid content, summarized in Table 1, confirms the marked difference between plant extracts, with nettle extract (AEUD) showing a 15.5‐fold higher content than carob extract (AECS) (31.18 ± 1.22 vs. 2.01 ± 0.47 mg QE/g). This difference is reflected in the slime composition: the control group (SSSD) shows 0.52 ± 0.018 mg QE/g, whereas nettle supplementation induces a significant and dose‐dependent increase at all doses compared to control (SSUD10% = 0.71 ± 0.064 mg QE/g, SSUD20% = 0.88 ± 0.067 mg QE/g, SSUD30% = 0.93 ± 0.082 mg QE/g), with values also significantly higher than carob groups at equivalent doses. Carob induces a significant increase only at 30% (SSCS30% = 0.63 ± 0.017 mg QE/g), whereas 10% and 20% doses do not differ from control (SSCS10% = 0.54 ± 0.026 mg QE/g, SSCS20% = 0.57 ± 0.009 mg QE/g). These results concord with total phenolics and confirm that plant‐based diet composition directly influences biochemical composition of slime, with a clearly superior effect of nettle.

### 3.4 Impact of Dietary Enrichment on Protein Composition of Snail Mucus

The protein content of the aqueous plant extracts is presented in Table 1. AEUD contains 23.66 ± 0.12 mg/g of dry matter, whereas AECS contains 8.31 ± 0.67 mg/g of dry matter. The standard snail group (SSSD) shows a protein content of 521 ± 1.57 mg/g of dry matter. Snails fed a nettle‐supplemented diet show a significant increase in mucus protein content compared with the SSSD group. The measured values were 605 ± 2.51 mg/g (SSUD10%), 615 ± 1.87 mg/g (SSUD20%), and 644 ± 1.49 mg/g (SSUD30%). Similarly, snails fed a carob‐supplemented diet show a significant increase in mucus protein content compared with the SSSD group, with values of 597 ± 1.24 mg/g (SSCS10%), 580 ± 3.67 mg/g (SSCS20%), and 568 ± 2.81 mg/g (SSCS30%). Overall, SSUD and SSCS groups show significantly higher protein contents than the SSSD group. In addition, SSUD groups tend to exhibit slightly higher protein values than SSCS groups at equivalent concentrations. A variation according to the level of dietary supplementation is also observed.

### 3.5 Variation of Sugar Content in Snail Mucus According to Dietary Supplementation

The sugar content of the aqueous extracts is presented in Table 1, which shows that AEUD contains 29.39 ± 0.061 mg/g of dry matter, whereas AECS exhibits a markedly higher content of 208.7 ± 0.092 mg/g of dry matter. Groups of snails fed a nettle‐supplemented diet showed a significant increase in mucus sugar content compared with the SSSD group. The measured values were 2.53 ± 0.058, 2.73 ± 0.87, and 2.81 ± 0.035 mg/g for SSUD10%, SSUD20%, and SSUD30%, respectively. A more pronounced significant increase was observed in snails fed a carob‐supplemented diet, with sugar contents of 4.42 ± 1.82, 4.86 ± 0.55, and 5.12 ± 0.57 mg/g for SSCS10%, SSCS20%, and SSCS30%, respectively. Overall, mucus sugar content was significantly higher in SSCS groups than in SSUD groups at corresponding supplementation levels, whereas both SSUD and SSCS groups showed significantly higher values compared with the SSSD group. A general increasing trend with the level of dietary supplementation was also observed.

### 3.6 In Vitro Antioxidant Capacity

The antioxidant activity, evaluated by the DPPH assay and expressed as 50% inhibitory concentration (IC_50_, μg/mL), shows that quercetin used as positive reference standard presents the highest activity (IC_50_ = 58.36 ± 0.31 μg/mL). Among the tested samples, standard slime (SSSD) displays notable intrinsic antioxidant activity (IC_50_ = 160.36 ± 0.32 μg/mL). Nettle supplementation induces a significant and dose‐dependent improvement in the antioxidant activity of slime at all doses compared to control (SSUD10% = 142.36 ± 0.26 μg/mL, SSUD20% = 136.19 ± 0.088 μg/mL, SSUD30% = 131.81 ± 0.17 μg/mL; all *p* < 0.005 vs. SSSD), with IC₅₀ values significantly lower than carob groups at equivalent doses, indicating the superior antioxidant capacity of nettle. Carob also improves antioxidant activity at all doses compared to control (SSCS10% = 156.4 ± 0.13 μg/mL, SSCS20% = 149.31 ± 0.21 μg/mL, SSCS30% = 141.55 ± 0.076 μg/mL). The most active slime is that from snails supplemented with 30% nettle (SSUD30%, IC_50_ = 131.81 μg/mL), whose activity remains 2.3‐fold lower than quercetin. The increase in antioxidant activity (lower IC_50_) directly corresponds to the increase in phenolic and flavonoid content: the samples richest in phenols (SSUD30%: 0.64 mg GAE/g) and flavonoids (SSUD30%: 0.93 mg QE/g) also present the lowest IC_50_ (131.81 μg/mL), confirming that these compounds are the main contributors to the antioxidant activity of the slime (Table 1).

### 3.7 Qualitative and Quantitative Macroscopic Evaluation of Anti‐Ulcer Activity

**FIGURE 1:**
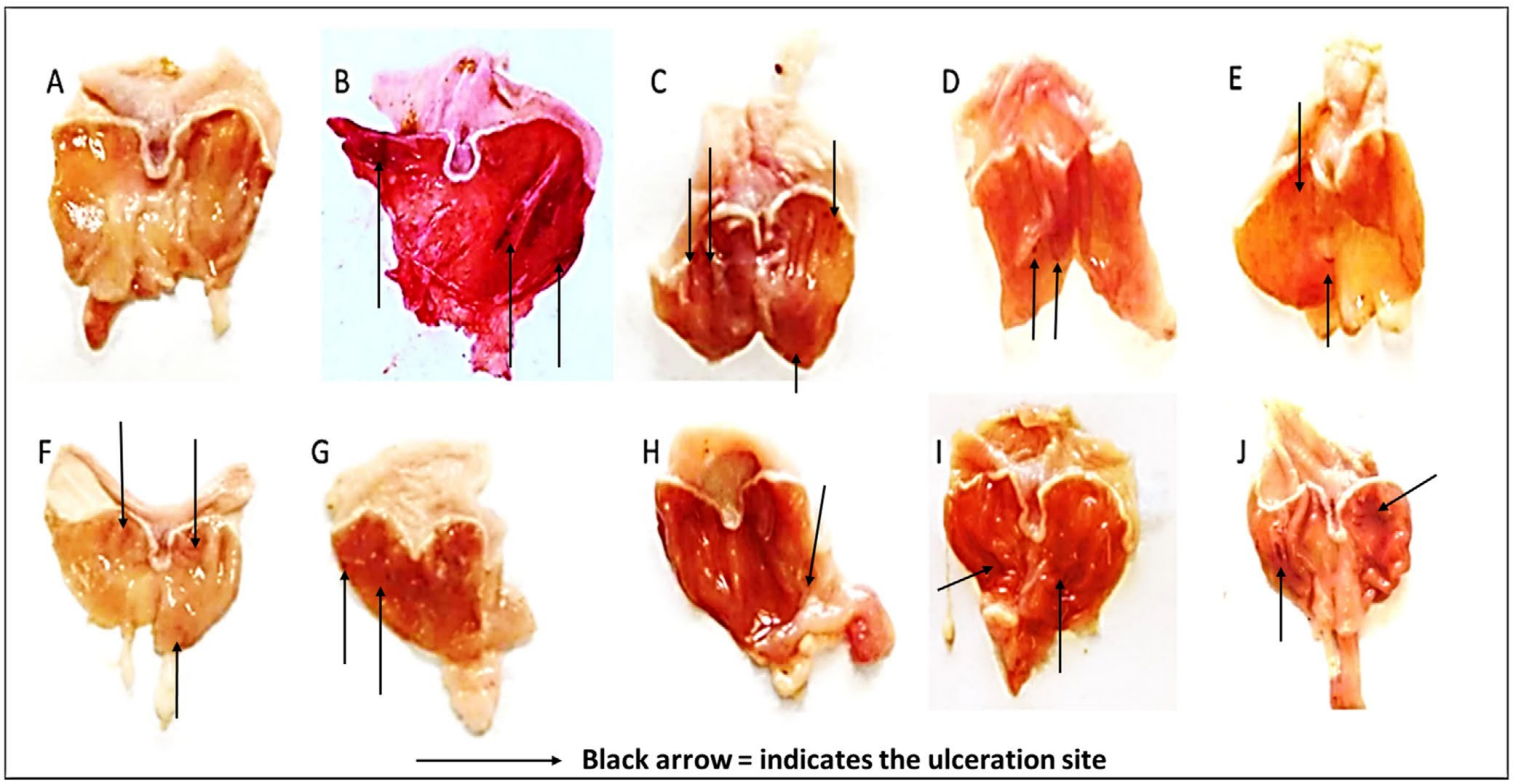
Gastric morphology showing the protective effects of snail mucus fed with different doses of nettle, carob, and famotidine (FAM) on ethanol‐induced ulcers. Animals were treated with doses of snail mucus (25 mg/kg, p.o.) and FAM (20 mg/kg, p.c., p.o.) or a vehicle (H_2_O). (A) H_2_O; (B) H_2_O + EtOH; (C) SSSD (25 mg/kg, p.o.) + EtOH; (D) SSUD10% (25 mg/kg, p.o.) + EtOH; (E) SSUD20% (25 mg/kg, p.o.) + EtOH; (F) SSUD30% (25 mg/kg, p.o.) + EtOH; (G) SSCS10% (25 mg/kg, p.o.) + EtOH; (H) SSCS20% (25 mg/kg, p.o.) + EtOH; (I) SSCS30% (25 mg/kg, p.o.) + EtOH; and (J) famotidine (20 mg/kg, p.c., p.o.).

The results presented in Table 2 show that ethanol administration markedly altered gastric parameters, as evidenced by a significant decrease in gastric pH to 2.11 ± 0.059 and a concomitant increase in gastric juice volume to 2.67 ± 0.096 mL/100 g compared with the control group (3.82 ± 0.094 and 1.56 ± 0.068 mL/100 g, respectively). Ethanol also induced extensive mucosal damage, with a mean ulcer area of 23.8 ± 1.06 mm^2^ (Figure 1B). Pre‐treatment with snail mucus obtained from animals fed a standard diet (SSSD, Figure 1C) or diets supplemented with nettle (SSUD 10%, 20%, and 30%; Figure 1D–F) or carob (SSCS 10%, 20%, and 30%; Figure 1G–I) significantly attenuated these ethanol‐induced changes, as reflected by a visible reduction in mucosal haemorrhagic lesions across the treated groups. All treated groups showed a significant increase in gastric pH and a significant reduction in gastric juice volume and ulcer area compared with the EtOH group. The most pronounced protection was observed in the SSUD30% group, which displayed the highest gastric pH (3.75 ± 0.074), the lowest gastric juice volume (1.59 ± 0.071 mL/100 g), and the smallest ulcer area (0.94 ± 0.012 mm^2^) (Figure 1F). Famotidine also improved gastric parameters, with values of 3.29 ± 0.078 for pH, 1.96 ± 0.082 mL/100 g for gastric volume, and 5.63 ± 0.078 mm^2^ (Figure 1J) for ulcer area, although its protective effect remained less marked than that of the highest‐dose snail mucus groups.

**TABLE 2:** Protective effects of snail mucus fed with standard diet, different doses of nettle, carob and famotidine (FAM) on ethanol‐induced disturbances in pH, gastric juice volume and ulcer area.

Pretreatment	pH of gastric juice	Gastric volume (mL/100 g)	Ulcer area (mm^2^)
Control	3.82 ± 0.094	1.56 ± 0.068	/
EtOH	2.11 ± 0.059*	2.67 ± 0.096*	23.8 ± 1.06*
EtOH + SSSD	3.37 ± 0.081^#^	1.91 ± 0.18^#^	2.01 ± 0.061^#^
EtOH + SSUD10%	3.61 ± 0.07^#^	1.73 ± 0.084^#^	1.72 ± 0.015^#^
EtOH + SSUD20%	3.71 ± 0.061^#^	1.63 ± 0.09^#^	1.58 ± 0.082^#α^
EtOH + SSUD30%	3.75 ± 0.074^#^	1.59 ± 0.071^#^	0.94 ± 0.012^#^
EtOH + SSCS10%	3.48 ± 0.055^#^	1.86 ± 0.08^#^	1.86 ± 0.067^#^
EtOH + SSCS20%	3.58 ± 0.096^#^	1.81 ± 0.064^#^	1.64 ± 0.053^#^
EtOH + SSCS30%	3.67 ± 0.062^#^	1.71 ± 0.058^#^	1.21 ± 0.027^#α^
EtOH + famotidine	3.29 ± 0.078^#^	1.96 ± 0.082^#^	5.63 ± 0.078^#^

*Note:* Animals were treated with doses of snail mucus (25 mg/kg. p.o.) and FAM (20 mg/kg. p.o.) or a vehicle (H_2_O). The data are expressed as mean ± SEM (*n* = 6).

*p < 0.05 compared to control group, ^#^ *p* < 0.05 compared to EtOH group, and ^α^ *p* < 0.05 compared to SSS.

### 3.8 Biochemical Measurements

The protective effects of snail mucus on ethanol‐induced plasma alterations are presented in Table 3. The evaluated biomarkers included markers of inflammation (CRP), hepatic function (AST, ALT, ALP, and LDH), pancreatic function (amylase), renal function (urea and creatinine), and lipid metabolism (HDL and LDL). Ethanol administration induced a significant increase in all measured plasma biomarkers compared with the control group, except for HDL, which was significantly decreased. CRP levels increased from 0.347 ± 0.016 mg/L in the control group to 1.11 ± 0.069 mg/L in the EtOH group. Similarly, the activities of amylase, AST, ALT, ALP, and LDH were significantly elevated in the EtOH group. Urea, creatinine, and LDL levels also increased, whereas HDL decreased from 0.71 ± 0.045 to 0.47 ± 0.023 mmol/L. Pretreatment with snail mucus significantly attenuated these disturbances compared with the EtOH group. A progressive improvement in parameters was observed with increasing concentrations of dietary supplements. The SSUD30% and SSCS30% groups showed the most pronounced effects, with CRP levels reduced to 0.368 ± 0.0076 and 0.431 ± 0.019 mg/L, respectively. In these groups, AST, ALT, ALP, LDH, and amylase activities were significantly lower than in the EtOH group and close to control values. Regarding renal markers, snail mucus treatments significantly reduced urea and creatinine levels compared with the EtOH group. The lowest values were observed in groups receiving the highest concentrations of snail mucus enriched with nettle or carob. Ethanol‐induced alterations in lipid profile were also improved by the treatments. A progressive increase in HDL and a decrease in LDL were observed in treated groups, particularly in SSUD30% and SSCS30%, whose values approached those of the control group. The SSSD group also showed significant improvements compared with the EtOH group, although these effects were generally less pronounced than those observed in groups receiving snail mucus from snails fed nettle‐ or carob‐enriched diets. Famotidine treatment (20 mg/kg) also significantly reduced ethanol‐induced alterations; however, for several parameters, the SSUD30% and SSCS30% groups showed values closer to those of the control group.

**TABLE 3:** Protective effects of snail mucus fed with standard diet, different doses of nettle, carob and famotidine (FAM) on ethanol‐induced disturbances in plasma analysis.

	Control	EtOH	SSSD	SSUD10%	SSUD20%	SSUD30%	SSCS10%	SSCS20%	SSCS30%	FAM
CRP (mg/L)	0.347 ± 0.016	1.11 ± 0.069*	0.533 ± 0.041*^#^	0.48 ± 0.034^#^	0.408 ± 0.0098^#^	0.368 ± 0.0076^#^	0.521 ± 0.012^#^	0.496 ± 0.0061^#^	0.431 ± 0.019^#^	0.456 ± 0.017^#^
Amylase (U/L)	258.5 ± 1.124	514 ± 2.011*	366.54 ± 2.06^#^	314.54 ± 1.54^#^	301.24 ± 1.88^#^	274.2 ± 0.67^#^	320.1 ± 0.47^#^	319.21 ± 1.45^#^	277.25 ± 0.87^#^	384.3 ± 1.28^#^
Urea (mmol/L)	4.36 ± 0.96	6.12 ± 0.64*	4.71 ± 0.51^#^	4.68 ± 0.87^#^	4.61 ± 0.24^#^	4.57 ± 0.22^#^	4.57 ± 0.82^#^	4.55 ± 0.24^#^	4.47 ± 0.22^#^	5.06 ± 0.15^#^
Creatinine (μmol/L)	36.11 ± 1.25	41.31 ± 1.45*	38.14 ± 1.54^#^	37.33 ± 1.47^#^	37.23 ± 1.54^#^	36.87 ± 1.57^#^	37.93 ± 1.47^#^	37.57 ± 1.56^#^	37.25 ± 0.85^#^	38.15 ± 1.87^#^
AST (U/L)	67.177 ± 1.22	137.33 ± 1.56*	71.87 ± 1.55^#^	71.67 ± 1.02^#^	70.45 ± 2.22^#^	68.36 ± 1.29^#^	71.72 ± 1.78^#^	70.84 ± 1.22^#^	69.24 ± 1.45^#^	70.04 ± 1.43^#^
ALT (U/L)	32.24 ± 1.62	55.45 ± 1.36*	32.46 ± 1.64^#^	31.87 ± 1.15^#^	31.58 ± 2.11^#^	31.35 ± 1.71^#^	32.22 ± 1.02^#^	31.74 ± 1.64^#^	31.61 ± 1.31^#^	32.81 ± 1.41^#^
ALP (U/L)	55.28 ± 1.67	112.71 ± 1.42*	56.81 ± 0.31^#^	56.45 ± 0.49^#^	55.78 ± 1.07^#^	55.52 ± 1.61^#^	56.87 ± 2.49^#^	56.45 ± 1.65^#^	55.78 ± 1.02^#^	55.97 ± 1.13^#^
LDH (U/L)	256.41 ± 1.41	451.2 ± 1.87*	324.74 ± 1.41^#^	287.81 ± 1.31^#^	277.7 ± 1.37^#^	264.3 ± 1.12^#^	311.2 ± 1.34^#^	281.61 ± 1.41^#^	273.41 ± 1.41^#^	281.5 ± 1.34^#^
HDL (mmol/L)	0.71 ± 0.045	0.47 ± 0.023*	0.58 ± 0.024^#^	0.61 ± 0.054^#^	0.65 ± 0.098^#^	0.68 ± 0.023^#^	0.65 ± 0.047^#^	0.65 ± 0.087^#^	0.66 ± 0.0313^#^	0.63 ± 0.061^#^
LDL (mmol/L)	0.30 ± 0.021	0.46 ± 0.079*	0.34 ± 0.036^#^	0.32 ± 0.036^#^	0.32 ± 0.0271^#^	0.31 ± 0.013^#^	0.33 ± 0.066^#^	0.33 ± 0.021^#^	0.32 ± 0.071^#^	0.31 ± 0.071^#^

*Note:* Animals were pretreated with various doses of snail mucus (25 mg/kg. p.o.) and FAM (20 mg/kg. p.o.) or a vehicle (H_2_O). The data are expressed as mean ± SEM (*n* = 6).

*p < 0.05 compared to the control group and ^#^ *p* < 0.05 compared to the EtOH.

### 3.9 Effects on Oxidative Stress Markers

**FIGURE 2:**
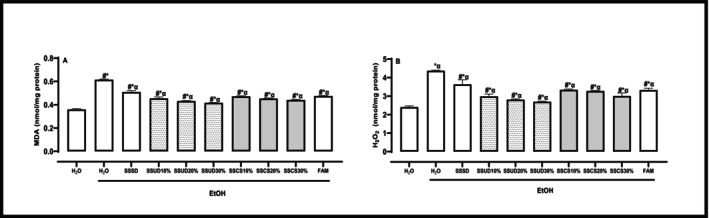
Effect of a pretreatment with different types of mucus SSSD, SSUD and SSCS (25 mg/kg BW, p.o.) or famotidine (20 mg/kg BW, p.o.) on the variation of the rate of MDA (A) and H_2_O_2_ (B) levels. Animals were pretreated with doses of nettle (25 mg/kg, p.o.), carob (25 mg/kg, p.o.), and famotidine (20 mg/kg, p.o., i.p.) or a vehicle (H_2_O). The data are expressed as mean ± SEM (*n* = 6). *p < 0.05 compared to the control group, ^#^ *p* < 0.05 compared to the EtOH group, and ^α^ *p* < 0.05 compared to SSSD.

Figure 2A shows the variations in malondialdehyde (MDA) levels, a marker of lipid peroxidation used to assess oxidative stress in the gastric mucosa. Ethanol administration caused a significant increase in MDA levels (0.611 ± 0.0078 nmol/mg protein) compared to the control group (0.35 ± 0.0023 nmol/mg protein), indicating substantial oxidative damage. In contrast, all snail mucus pretreatments (SSSD, SSUD, and SSCS) significantly reduced this elevation. The most pronounced effects were observed with SSUD and SSCS, with values close to those of the control group, whereas SSSD showed a more moderate protective effect. Famotidine also reduced MDA levels (0.479 ± 0.0083 nmol/mg protein), but its effect was less pronounced than that of some of the tested extracts.

Figure 2B presents the results for hydrogen peroxide (H_2_O_2_). Ethanol administration significantly increased its concentration in gastric tissue (4.358 ± 0.012 nmol/mg protein) compared to the control group (2.671 ± 0.0082 nmol/mg protein), confirming marked oxidative stress induction. Pretreatment with snail mucus extracts (SSSD, SSUD, and SSCS) significantly decreased H_2_O_2_ levels. SSUD and SSCS, particularly at 20% and 30% concentrations, showed stronger effects, with values approaching those of the control group. Famotidine also reduced H_2_O_2_ levels (3.335 ± 0.0095 nmol/mg protein), although its effect remained generally lower than that of the most active extracts.

### 3.10 Antioxidant Enzyme Activities

**FIGURE 3:**
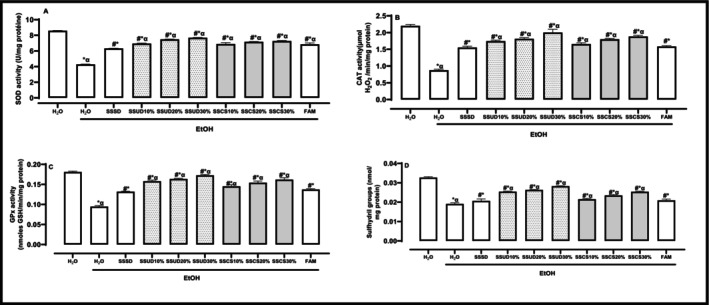
Effect of a pretreatment with different types of mucus SSSD, SSUD and SSCS (25 mg/kg BW, p.o.) or famotidine (20 mg/kg BW, p.o.) on the variation of the rate of: SOD (A), CAT (B), and GPx (C), as well as the non‐enzymatic activity of thiol groups (D). Animals were pretreated with doses of nettle (25 mg/kg, p.o.), carob (25 mg/kg, p.o.), and famotidine (20 mg/kg, p.o., i.p.) or a vehicle (H_2_O). The results are expressed as mean ± SEM (*n* = 6). *p < 0.05 compared to the control group, ^#^ *p* < 0.05 compared to the EtOH group, and ^α^ *p* < 0.05 compared to SSSD.

Ethanol administration induced a marked impairment of the antioxidant system, as evidenced by significant decreases in the enzymatic activities of superoxide dismutase (SOD, U·mg^−1^ protein) (Figure 3A), catalase (CAT, μmol H_2_O_2_·min^−1^·mg^−1^ protein) (Figure 3B), and glutathione peroxidase (GPx, nmol GSH·min^−1^·mg^−1^ protein) (Figure 3C) compared with the control group. Specifically, SOD activity decreased from 8.575 ± 0.018 to 4.295 ± 0.240 (U·mg^−1^ protein) in the EtOH group. CAT activity declined from 2.12 ± 0.048 to 0.85 ± 0.024 (μmol H_2_O_2_·min^−1^·mg^−1^ protein), whereas GPx activity decreased from 0.181 ± 0.027 to 0.092 ± 0.001 (nmol GSH·min^−1^·mg^−1^ protein), confirming significant ethanol‐induced oxidative stress. Snail mucus pretreatment significantly restored antioxidant enzyme activities compared with the EtOH group. The most pronounced effects were observed in the SSUD30% and SSCS30% groups, where SOD activities reached 7.710 ± 0.360 and 7.310 ± 0.360 (U·mg^−1^ protein), respectively. Similarly, CAT activity was restored to 2.11 ± 0.063 (μmol H_2_O_2_·min^−1^·mg^−1^ protein) (SSUD30%) and 1.92 ± 0.460 (μmol H_2_O_2_·min^−1^·mg^−1^ protein) (SSCS30%). GPx activity also increased, reaching 0.175 ± 0.061 (nmol GSH·min^−1^·mg^−1^ protein) for SSUD30% and 0.161 ± 0.051 (nmol GSH·min^−1^·mg^−1^ protein) for SSCS30%. These values approached those of the control group, indicating substantial restoration of the antioxidant defense system. Furthermore, the SSUD and SSCS groups exhibited significantly higher enzyme activities than the SSSD group, suggesting that dietary supplementation of snails with nettle leaves (SSUD) or carob (SSCS) enhances the antioxidant properties of snail mucus. The activities observed in the SSUD30% and SSCS30% groups also exceeded those of the famotidine‐treated group (FAM), highlighting the superior efficacy of these treatments in protecting against ethanol‐induced oxidative stress.

### 3.11 Sulfhydryl Group Concentration

Ethanol administration significantly decreased sulfhydryl (−SH) group levels in gastric tissue, indicating oxidative damage to thiol‐containing proteins. Pre‐treatment with snail slime extracts, particularly SSUD30%, restored −SH levels to near‐normal values, comparable to those observed with famotidine (Figure 3D). This further underscores the antioxidant and protective effects of snail slime.

### 3.12 Histological Evaluation

**FIGURE 4:**
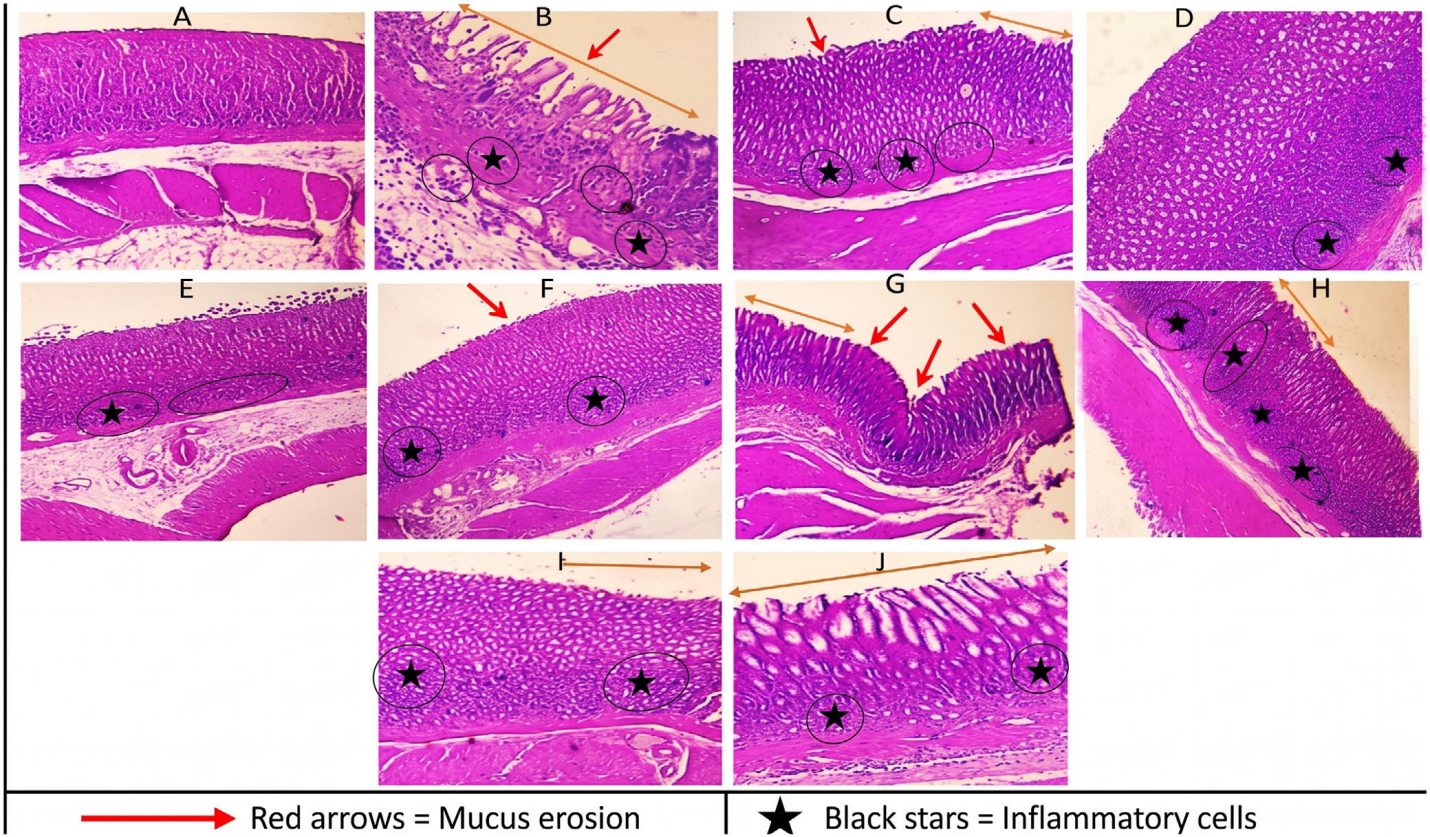
Gastric histology showing the protective effect of snail mucus fed with different doses of nettle, carob, and famotidine (FAM) on ethanol‐induced histological alterations in the stomach. The animals were treated with doses of snail mucus (25 mg/kg, p.o.) and FAM (20 mg/kg, p.c., p.o.) or vehicle (H_2_O). (A) H_2_O; (B) H_2_O + EtOH; (C) SSSD (25 mg/kg, p.o.) + EtOH; (D) SSUD10% (25 mg/kg, p.o.) + EtOH; (E) SSUD20% (25 mg/kg, p.o.) + EtOH; (F) SSUD30% (25 mg/kg, p.o.) + EtOH; (G) SSCS10% (25 mg/kg, p.o.) + EtOH; (H) SSCS20% (25 mg/kg, p.o.) + EtOH; (I) SSCS30% (25 mg/kg, p.o.) + EtOH; and (J) Famotidine (20 mg/kg, p.c., p.o.).

Histological examination of gastric tissues revealed marked lesions in ethanol‐treated rats, including epithelial disorganization, edema, hemorrhage, and inflammatory cell infiltration, compared with the intact gastric mucosa observed in the control group (Figure 4A). The ethanol‐treated group (Figure 4B) showed extensive mucus erosion (red arrows) and dense inflammatory cell infiltration (black stars), confirming severe gastric damage.

To provide a more objective assessment of these alterations, a histological damage score (HDS) was applied, as summarized in Table 4. Ethanol administration resulted in a high HDS (11), confirming severe gastric injury. Pretreatment with famotidine (Figure 4J) partially reduced histological lesions (HDS = 7; protection = 36.3%), with residual signs of mucus erosion and inflammatory infiltration still visible. In contrast, snail mucus‐based treatments markedly improved gastric architecture. The SSSD group (Figure 4C) reduced the HDS to 4 (63.64% protection), whereas SSUD and SSCS treatments showed dose‐dependent protective effects. SSUD reduced the HDS to 4 at 10% (Figure 4D), and further to 2 at 20% (Figure 4E) and 30% (Figure 4F), corresponding to 81.82% protection, with markedly reduced inflammatory cell presence and preserved epithelial architecture. Similarly, SSCS reduced the HDS to 4 at 10% (Figure 4G) and 20% (Figure 4H), and to 3 at 30% (Figure 4I) (72.73% protection).

**TABLE 4:** Protective effects of snail mucus obtained from snails fed a standard diet and different doses of nettle and carob on ethanol‐induced gastric lesions: Histological damage score (HDS) and percentage of protection.

Pretreatment	Epithelium	Edema	Hemorrhage	Inflammation	Total (HDS)	Protection (%)
Control	0	0	0	0	0	/
EtOH	3	3	2	3	11	/
EtOH + SSSD	1	1	1	1	4	63.64
EtOH + SSUD10%	1	1	1	1	4	63.64
EtOH + SSUD20%	1	0	0	1	2	81.82*
EtOH + SSUD30%	1	0	0	1	2	81.82*
EtOH + SSCS10%	1	1	1	1	4	63.64
EtOH + SSCS20%	1	1	1	1	4	63.64
EtOH + SSCS30%	1	1	0	1	3	72.73*
EtOH + famotidine	2	2	1	2	7	36.3

*Note:* Animals were pretreated with various doses of snail mucus (25 mg/kg p.o.) and FAM (20 mg/kg p.o.) or a vehicle (H_2_O). The data are expressed as mean ± SEM (*n* = 6).

*p < 0.05 compared to SSSD.

Overall, the histological sections presented in Figure 4 confirm the quantitative findings, showing markedly greater protection in the SSUD and SSCS groups compared with famotidine and SSSD. These results indicate that SSUD and SSCS treatments exhibit the strongest gastroprotective effects, particularly at higher concentrations, as evidenced by lower histological damage scores and higher protection percentages.

## 4 Discussion

The present study demonstrates that dietary supplementation of *H. aspersa* with nettle ( *U. dioica* ) or carob ( *C. siliqua* ) significantly modifies the biochemical composition of the secreted mucus (SSUD, SSCS) and enhances its gastroprotective efficacy against ethanol‐induced gastric lesions. Compared with mucus obtained from snails fed a standard diet (SSSD), mucus from supplemented groups exhibited higher concentrations of phenolic compounds, flavonoids, total proteins, and total sugars, together with greater in vitro antioxidant activity. These dose‐dependent effects are directly related to the phytochemical richness of the plants incorporated into the diet, confirming that snail nutrition is a key determinant of the biological activity of its mucus. This finding is consistent with previous studies demonstrating the protective effects of *H. aspersa* mucus or secretion filtrate against ethanol‐induced gastric ulcers through attenuation of oxidative stress and inflammatory damage (Petrov et al. 2022; Gugliandolo et al. 2021).

Dietary supplementation influenced the extraction yield of both plant extracts and snail mucus. The aqueous extracts of *U. dioica* and *C. siliqua* showed yields of 12.18% ± 0.97% and 9.47% ± 1.08% (w/w), respectively. Regarding snail mucus, significantly higher yields were observed in supplemented groups compared with the standard diet group (SSSD: 1.20% ± 0.07% w/w), with the highest values recorded in SSUD30% (2.82% ± 0.06%) and SSCS30% (2.51% ± 0.01%). These findings indicate that dietary plant supplementation modulates both mucus yield and dry matter recovery, likely reflecting changes in glandular secretion and mucus composition.

Comparative analysis of the plant extracts revealed substantial differences between the two species, which directly explain the variations observed in mucus composition. The aqueous nettle extract (AEUD) exhibited a phenolic content approximately 33‐fold higher than that of the aqueous carob extract (AECS) (218.5 ± 0.36 vs. 6.52 ± 0.59 mg GAE/g) and a flavonoid content 15.5‐fold higher (31.18 ± 1.22 vs. 2.01 ± 0.47 mg QE/g). This remarkable phytochemical richness of nettle is attributable to its well‐documented composition, including a wide range of polyphenols and flavonoids, particularly quercetin and rutin, as well as sterols and lignans with potent antioxidant and anti‐inflammatory properties (Fatima et al. 2018; Sadgrove et al. 2024). Although less rich in polyphenols, carob also possesses established gastroprotective properties attributed to its tannins, dietary fibers, and phenolic compounds, which exhibit anti‐inflammatory, antimicrobial, anti‐ulcer, and antioxidant activities (Rtibi et al. 2015).

These phytochemical differences were faithfully reflected in the secreted mucus. The SSSD group showed significantly lower phenolic content than the SSUD and SSCS groups, with nettle exerting a markedly greater effect across all tested doses. These findings confirm that bioactive compounds ingested by the snails are effectively incorporated into and transferred through the mucus, and that the phenolic composition of the dietary plant directly determines the biochemical enrichment of the mucus.

This phenolic enrichment was directly associated with enhanced in vitro antioxidant capacity of the mucus, establishing a functional link between biochemical composition and protective efficacy. Antioxidant activity, assessed by the DPPH assay and expressed as IC_50_, improved in a dose‐dependent manner with nettle supplementation. The SSUD30% group exhibited the highest antioxidant activity, with significantly lower IC_50_ values than the corresponding carob groups. The direct correlation between the higher phenolic (0.64 mg GAE/g) and flavonoid (0.93 mg QE/g) contents and the lowest IC_50_ value (131.81 μg/mL) confirms that these compounds are the major contributors to mucus antioxidant activity. Quercetin, a major flavonoid in nettle, is a potent scavenger of reactive oxygen species (ROS) and an effective inhibitor of pro‐inflammatory mediators such as TNF‐α and IL‐1α (Sadgrove et al. 2024; Valdés et al. 2016). These observations are consistent with recent studies highlighting the role of dietary polyphenols in strengthening gastric mucosal defense mechanisms through free radical scavenging and inhibition of pro‐inflammatory pathways (Cummings and Olsen 2011).

Beyond its intrinsic antioxidant capacity, dietary supplementation also induced a significant increase in total proteins and total sugars in the SSUD and SSCS groups compared with the SSSD group. This biochemical modification plays a fundamental role in gastroprotection by strengthening the physical and structural properties of mucus.

The increase in protein content reflects an overall enrichment of mucus with functional and bioactive proteins, particularly mucin‐type glycoproteins, which constitute the main structural components of the mucus gel (Molek et al. 1997; Laine et al. 2008). These highly glycosylated proteins determine the rheological and protective properties of mucus. Therefore, the higher protein content detected in supplemented groups may indicate a greater abundance of mucins, enhancing the mucosal barrier and resistance to ethanol‐induced injury. However, total protein quantification does not allow the specific identification of mucins. Therefore, this interpretation should be regarded as mechanistic but inferential. Future studies should include mucin‐specific assays or glycoproteomic analyses to confirm this hypothesis.

Moreover, *H. aspersa* mucus contains collagen and elastin, whose protective roles against gastric mucosal damage and inflammation have been documented (Gugliandolo et al. 2021; Ellijimi et al. 2018). These extracellular matrix proteins contribute to mucosal integrity and elasticity and promote the repair of damaged epithelial barriers (Ellijimi et al. 2018; Sarkar et al. 2025). Consequently, the increase in total proteins observed in supplemented groups likely reflects a combined enrichment in mucins and structural proteins, thereby reinforcing mucus resistance to ethanol‐induced aggression.

The increase in total sugar content, which was more pronounced in the carob groups (SSCS30%) due to the naturally higher carbohydrate content of this plant, likely reflects enhanced glycosylation of mucus components. Since mucins are highly glycosylated molecules, a higher total sugar content indicates a greater abundance of glycoprotein components contributing to mucus cohesion, viscosity, and hydration (Montalto et al. 2002).

The increased viscosity of enriched mucus represents one of the most direct protective mechanisms against ethanol‐induced lesions. By coating the gastric wall, mucus forms a protective layer between ingested ethanol and the epithelium, limiting direct contact between the damaging agent and the mucosa. This barrier function is well established: gastric mucus constitutes the only identifiable physical barrier between the gastric lumen and the mucosal surface, and its protective capacity depends directly on its viscoelastic and permselective properties. Any weakening of these properties is associated with impaired protection and the development of gastric diseases (Allen and Flemström 2005; Atuma et al. 2001). However, since mucus viscosity was not measured directly, this conclusion remains inferential and should be interpreted with caution.

The simultaneous increase in gastric pH (from 2.11 ± 0.059 in the EtOH group to 3.75 ± 0.074 in the SSUD30% group) and reduction in gastric juice volume (from 2.67 ± 0.096 to 1.59 ± 0.071 mL/100 g body weight) directly reflect this physical protection. The viscous mucus reduces ethanol penetration and partially neutralizes acidity at the epithelial surface, thereby attenuating direct tissue damage (Laine et al. 2008). These effects, which were more pronounced at higher nettle and carob concentrations and exceeded those achieved by famotidine, highlight the superiority of diet‐enriched mucus over a conventional reference treatment.

However, the physical protection provided by mucus viscosity alone cannot fully explain the observed effects and should be considered synergistically with antioxidant and anti‐inflammatory mechanisms. Ethanol induces gastric injury not only through direct epithelial damage but also through activation of oxidative and inflammatory cascades. At the molecular level, excessive ROS generation stimulates macrophages to release pro‐inflammatory mediators, including NF‐κB and TNF‐α, thereby aggravating tissue injury.

In the present study, ethanol administration significantly increased MDA and H_2_O_2_ levels, confirming severe oxidative stress in gastric tissue. Simultaneously, the activities of antioxidant enzymes SOD, CAT, and GPx were significantly reduced, indicating a collapse of endogenous defense systems. Pretreatment with enriched mucus restored these parameters in a dose‐dependent manner, with the most pronounced effects observed in the SSUD30% group, whose SOD, CAT, and GPx values approached those of the control group and exceeded those of the famotidine‐treated group. This enzymatic restoration is consistent with the activation of antioxidant defenses through the Nrf2/HO‐1 pathway, together with the concurrent inhibition of NF‐κB‐mediated inflammatory signaling by the flavonoids and polyphenols present in the enriched mucus (Khan et al. 2020; Valdés et al. 2016; Petrov et al. 2022). Notably, the NF‐κB and Nrf2 pathways are recognized as key regulators of the balance between inflammation and antioxidant defense. Although these pathways were not directly assessed in the present study, the observed biochemical profile strongly supports their likely involvement in the gastroprotective effects of the enriched mucus. Future studies should therefore include molecular analyses targeting inflammatory cytokines, COX‐2, and the NF‐κB and Nrf2 pathways to confirm the underlying mechanisms involved.

This antioxidant protection was directly reflected in systemic biomarkers and tissue architecture. The EtOH group exhibited significant increases in CRP, hepatic enzymes (AST, ALT, ALP, LDH), pancreatic amylase, renal markers (urea and creatinine), and LDL levels, together with a reduction in HDL, indicating that ethanol‐induced injury extended well beyond the gastric mucosa. Pretreatment with enriched mucus significantly attenuated all these alterations in a dose‐dependent manner. The SSUD30% group exhibited the most pronounced effects, with CRP reduced to 0.368 ± 0.0076 mg/L and lipid profile parameters approaching control values, confirming that enriched mucus exerts not only local gastroprotective effects but also systemic anti‐inflammatory activity (Petrov et al. 2022; Gugliandolo et al. 2021).

Histologically, ethanol administration resulted in a histological damage score (HDS) of 11, corresponding to severe epithelial disorganization, edema, hemorrhage, and inflammatory cell infiltration. Famotidine reduced this score only to 7. In contrast, SSUD20% and SSUD30% reduced the HDS to 2 (81.82% protection), whereas SSCS30% reduced it to 3, demonstrating markedly greater histoprotective efficacy than the reference drug.

Taken together, these findings support a comprehensive gastroprotective model based on three complementary and interdependent mechanisms. First, phenolic and flavonoid enrichment of mucus, directly determined by snail dietary composition, enhances its intrinsic antioxidant capacity and preserves the structural integrity of glycoprotein components against oxidative degradation. Second, this preservation maintains the viscosity and rheological properties of the mucus gel, which constitutes the first line of physical defense against ethanol penetration, reinforced by the simultaneous increase in total proteins and total sugars. Third, flavonoids and polyphenols incorporated into the mucus exert direct antioxidant and anti‐inflammatory effects upon contact with the gastric mucosa through modulation of the Nrf2/HO‐1 and NF‐κB pathways, restoring endogenous enzymatic defenses and reducing systemic inflammatory cascades.

The combination and interdependence of these three mechanisms physical barrier formation, antioxidant protection, and anti‐inflammatory modulation explain the superior performance of the SSUD30% group across all evaluated parameters and support the optimization of snail mucus through dietary supplementation as a promising strategy for the development of natural gastroprotective agents.

## 5 Conclusion

In conclusion, this study demonstrates that dietary supplementation of *H. aspersa* with nettle ( *U. dioica* ) and carob ( *C. siliqua* ) significantly improves the biochemical composition and gastroprotective properties of snail mucus. Enriched mucus exhibited higher levels of phenolic compounds, flavonoids, proteins, and sugars, which were associated with enhanced antioxidant activity. Dietary supplementation also increased the lyophilised mucus yield, particularly in the nettle‐supplemented groups, indicating an improvement in the recovery of mucus dry matter in addition to its biochemical enrichment. Mucus obtained from snails fed a diet enriched with 30% nettle (SSUD30%) showed the most pronounced effects, reducing gastric lesions, oxidative stress, and inflammation while preserving gastric mucosal integrity. These effects appear to result from a combined action involving reinforcement of the mucus barrier, enhancement of antioxidant defenses, and modulation of the inflammatory response. These findings highlight the potential of enriched snail mucus, particularly that obtained from nettle‐fed snails, as a promising natural source of gastroprotective agents. However, further studies are required to identify the specific bioactive compounds involved and to confirm its therapeutic potential in humans.

## Author Contributions

**Karima Tlili:** methodology. **Hanen Kahlaoui:** methodology. **Khaoula Gharbi:** conceptualization, investigation, writing – original draft, methodology, visualization, software, formal analysis, project administration, data curation, supervision, resources, validation, funding acquisition. **Slimen Selmi:** visualization, validation, conceptualization, writing – review and editing. **Soumaya Wahabi:** methodology. **Hichem Sebai:** writing – review and editing, methodology. **Stefano D'allacqua:** methodology, data curation.

## Funding

The authors have nothing to report.

## Ethics Statement

All procedures on animals in this study were compiled with the National Institute of Health recommendations for the use and care of animals. The study was conducted according to the guidelines of the Declaration of Helsinki and approved by the Institutional Review Board at the University of Jendouba, Tunisia.

## Conflicts of Interest

The authors declare no conflicts of interest.

## Data Availability

The data that support the findings of this study are available from the corresponding author upon reasonable request.
